# A Bioinformatics Approach for Determining Sample Identity from Different Lanes of High-Throughput Sequencing Data

**DOI:** 10.1371/journal.pone.0023683

**Published:** 2011-08-17

**Authors:** Rachel L. Goldfeder, Stephen C. J. Parker, Subramanian S. Ajay, Hatice Ozel Abaan, Elliott H. Margulies

**Affiliations:** Genome Informatics Section, Genome Technology Branch, National Human Genome Research Institute, National Institutes of Health, Bethesda, Maryland, United States of America; Tel Aviv University, Israel

## Abstract

The ability to generate whole genome data is rapidly becoming commoditized. For example, a mammalian sized genome (∼3Gb) can now be sequenced using approximately ten lanes on an Illumina HiSeq 2000. Since lanes from different runs are often combined, verifying that each lane in a genome's build is from the same sample is an important quality control. We sought to address this issue in a post hoc bioinformatic manner, instead of using upstream sample or “barcode” modifications. We rely on the inherent small differences between any two individuals to show that genotype concordance rates can be effectively used to test if any two lanes of HiSeq 2000 data are from the same sample. As proof of principle, we use recent data from three different human samples generated on this platform. We show that the distributions of concordance rates are non-overlapping when comparing lanes from the same sample versus lanes from different samples. Our method proves to be robust even when different numbers of reads are analyzed. Finally, we provide a straightforward method for determining the gender of any given sample. Our results suggest that examining the concordance of detected genotypes from lanes purported to be from the same sample is a relatively simple approach for confirming that combined lanes of data are of the same identity and quality.

## Introduction

Recent advances in sequencing throughput offer the ability to generate useful data from multiple individuals on a single run. For example, on the Illumina platform, multiple samples are typically separated into different lanes, and then combined together based on sample identity [1]. Other technologies have similar approaches to running multiple samples on a single run [2], [3]. While methods exist to “barcode” samples to confirm the downstream identity of the generated sequences [4]–[6], this requires significant modifications to sample preparation methods and is more appropriate for pooling multiple samples in the same lane (so-called “multiplexing”). Currently, no independent methods have been published to computationally determine if two lanes of data are from the same sample.

We sought to develop a validation approach that could be used without any upstream sample preparation modifications. The impetus for this work came from our increased use of the newly developed HiSeq 2000, which allows us to generate high-coverage (30X or greater) whole-genome data with approximately 10 lanes. This means we frequently run more than one human sample on a flowcell. Additionally, the Illumina workflow introduces a “flowcell flip” between the cBot cluster station and the HiSeq 2000 sequencing instrument, which requires samples to be initially loaded on to the flowcell in reverse order. This confusing and potentially error-prone step, combined with the ability to run two flowcells at once, increases the importance of verifying the identity of combined lanes, especially when a flowcell contains more than one sample from the same species (and thus simple reference genome alignment statistics cannot verify flowcell orientation).

Our approach relies on the inherent diversity in the human population; unrelated humans vary in roughly 1 out of 1000 bases [1], [7]. We propose that a sufficient number of genotype calls can be made with data from a single HiSeq 2000 lane, resulting in enough overlapping positions between any two lanes of data to produce an accurate concordance rate. Two lanes from the same individual should have a relatively high concordance rate, while two lanes from different individuals should have a lower concordance rate. We suggest that the difference in concordance rates can be used to accurately determine if two lanes of data are from the same library and of comparable quality.

In this paper, we show that there are enough data in a single HiSeq 2000 lane to produce genotype concordance rates between lanes from the same sample that do not overlap with concordance rates between lanes from different samples. We illustrate our findings by analyzing 24 lanes of HiSeq 2000 data from three different human samples. Our method proves to be robust even when different numbers of reads are analyzed. Finally, we also provide a straightforward method for determining the gender of a given sample.

## Results

Before looking at the similarity of polymorphisms within the autosomes, one must first determine if the data from different lanes are from humans of the same gender. For this we identified a straightforward approach that examines the depth of coverage at specific “well-behaved” regions on the X and Y chromosomes (see Methods). Once the predicted gender matches between different lanes, a more robust and sensitive approach can then be applied to determine if the data from different lanes came from the same sample.

**Figure 1:**
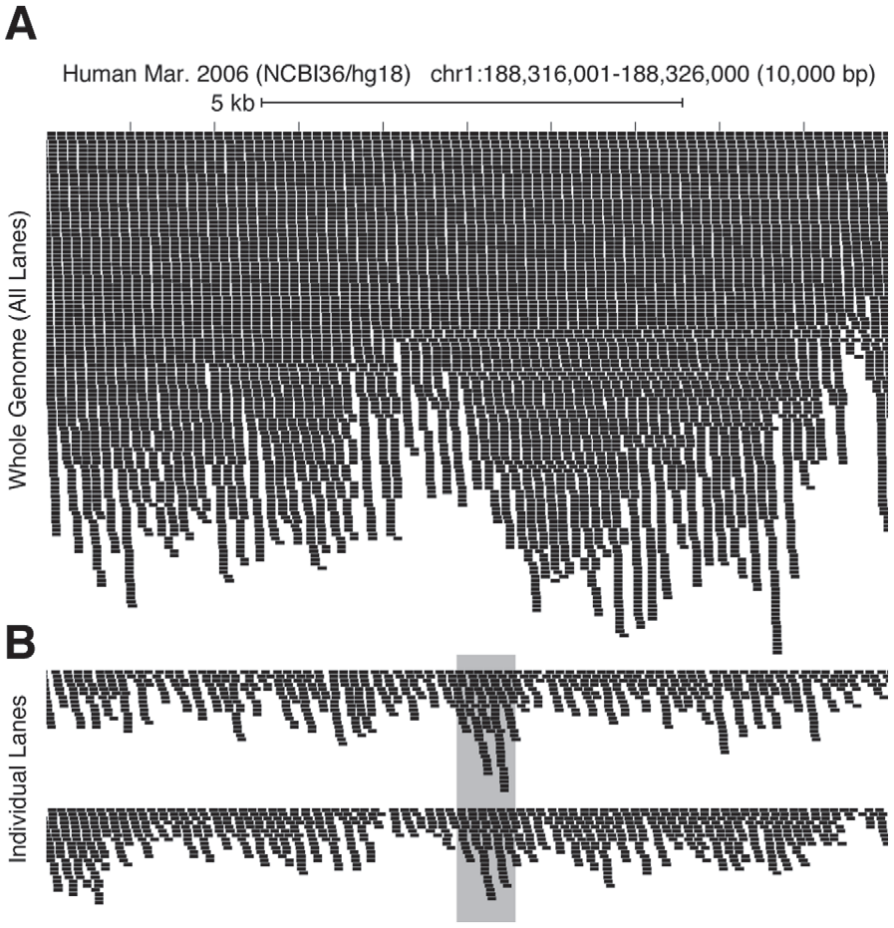
Overview of approach. Several lanes of HiSeq 2000 data are typically combined together for a comprehensive genome analysis, giving a high depth of coverage (A), and the ability to accurately call genotypes in the majority of the genome. In (B), two individual lanes of HiSeq 2000 data are depicted, with a lower average depth of coverage. By chance, some regions of the genome have enough data to be genotyped in both lanes (shaded gray).

We first wanted to determine if there were sufficient data from a typical (circa fall 2010) single lane of HiSeq 2000 data to call genotypes in a subset of the human genome. Based on the Lander-Waterman statistics [8], we do not expect completely uniform read coverage across the genome, but rather there will likely be small regions of the genome with sufficient coverage for accurately calling genotypes (see Figure 1). Indeed, from 24 lanes across three flowcells, we were able to call an average of 233 million autosomal genotypes per lane (see Table 1). There were also sufficient data to unambiguously determine the gender of each sample.

**Table 1:** Summary of data used in these analyses.

Sample ID	Lane	Number of Reads	Number of Genotypes called	Gender
A	1	113,453,840	20,184,635	Male
A	2	137,855,285	63,745,441	Male
A	3	136,937,966	60,964,182	Male
A	4	138,649,724	62,318,000	Male
A	5	140,173,232	68,523,311	Male
A	6	139,484,272	66,008,548	Male
A	7	138,052,195	63,689,638	Male
A	8	137,106,804	61,172,939	Male
B	1	183,082,811	333,308,543	Male
B	2	184,740,055	342,648,944	Male
B	3	183,494,075	334,034,308	Male
B	4	185,367,578	345,182,564	Male
B	5	184,384,025	342,715,958	Male
B	6	182,873,463	329,476,404	Male
B	7	185,063,316	345,695,140	Male
B	8	186,383,475	359,973,464	Male
C	1	186,723,675	305,090,551	Female
C	2	186,823,494	306,386,070	Female
C	3	186,767,296	303,986,501	Female
C	4	183,046,388	274,637,962	Female
C	5	185,501,762	296,607,392	Female
C	6	185,939,130	295,223,049	Female
C	7	187,938,913	311,994,826	Female
C	8	186,314,862	302,012,031	Female

Number of reads reflects the number of aligning reads after removing duplicate read pairs and filtering for low quality alignments (see Methods). Gender was determined by looking at coverage of reads in specific representative regions of the X and Y chromosomes (see Methods). Number of genotypes called is from the autosomes only, which is what was used for downstream comparisons.

Using these datasets, we wanted to test the hypothesis that there exist a sufficient number of genotype comparisons between two different lanes to distinguish higher concordance rates, which result from lanes with the same sample, versus lower concordance rates, which result from lanes with different samples. To do this, we performed an all-by-all pair-wise lane comparison of genotypes; this allowed us to generate distributions of concordance rates for multiple same- and different-sample comparisons. For this experiment, we took all 24 lanes from the three flowcells listed in Table 1, and performed 276 pair-wise lane comparisons. On average, there were 42 million positions compared between any two lanes.

**Figure 2:**
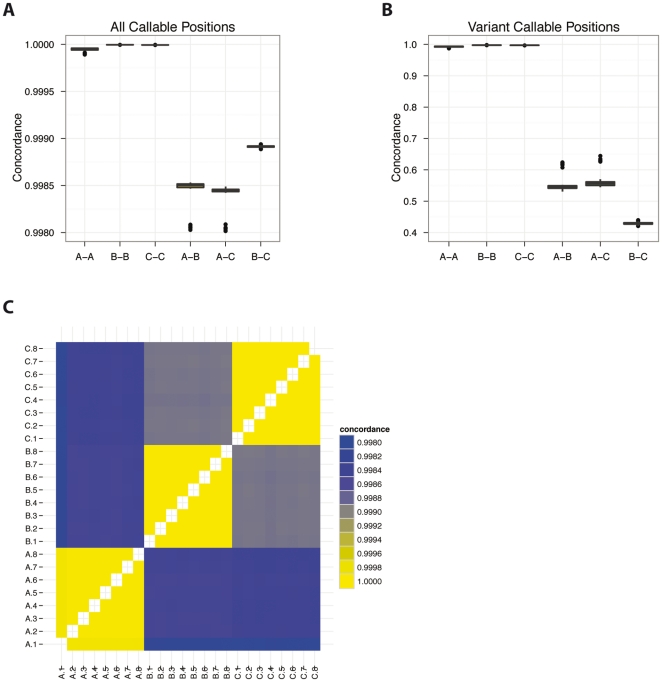
Concordance between lanes. Distributions of genotype concordance rates from same- and different-sample comparisons are non-overlapping. The box plot in (A) shows the distributions of concordance rates when using all callable positions for all combinations of pairs of the three samples being analyzed. The x-axis denotes each pair being compared (A, B, and C, refer to the sample IDs in Table 1), and the y-axis represents the distribution of concordance rates for all pair-wise combinations of lanes representing the specific pair of samples on the x-axis. It is likely that the detected differences from same-sample comparisons (B–B, C–C, and A–A) arise solely from sequencing and genotyping error. The box plot in (B) is similar to (A), except that here only variant (nonreference) positions are considered. The symmetrical heat map in (C) summarizes the data from panel (A); the blue boxes represent low concordance rates and correspond to different-sample comparisons, while the yellow boxes along the diagonal represent high concordance rates and correspond to same-sample comparisons. Note that comparisons between samples B and C (gray boxes) are slightly more similar to each other than the other different-sample comparisons, but still sufficiently distinct from same-sample comparisons. This is expected given the known partial consanguinity between these individuals.

The results of this analysis support our hypothesis that concordance rates can be used to distinguish same-sample versus different-sample comparisons with single lanes of HiSeq 2000 data (Figure 2). Importantly, data from each type of comparison have tight non-overlapping distributions when considering all callable positions or variant (nonreference) callable positions (Figures 2A and B). Same-sample lane comparisons have the highest concordance rates and between-sample comparisons have the lowest concordance rates. Interestingly, the all-positions concordance rates between samples B and C have a distinct non-overlapping distribution that is slightly more concordant than the other two different-sample comparisons (A–B and A–C) (Figure 2A), which reflects the partial consanguinity (likely going back > 6 generations) that exists between these two individuals (T. Markello, personal communication). These results are also illustrated as a heat map (Figure 2C), which nicely distinguishes same- versus different-sample comparisons.

**Figure 3:**
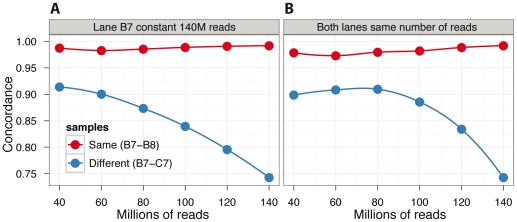
Effect of data quantity on concordance rates. The total number of reads used in the analysis affects different-sample comparisons, but not same-sample comparisons. In (A), lane 7 of sample ID B was kept constant at 140 million reads (B7), and the amount of data for the other sample [either lane 8 of B (B8) or lane 7 of C (C7)] was varied between 40 million and 140 million reads (x-axis) in 20 million read increments. The y-axis represents the concordance rate between variant (nonreference) genotypes called between the two different datasets. Note that for the same-sample comparison (red line), varying the number of reads used in the analysis does not substantially alter the concordance rate. However, this is not the case for different-sample comparisons (blue line), where the concordance rate becomes more different as more reads are used. In (B), a similar trend is observed when the reads in both samples are incremented simultaneously. Solid lines represent a LOESS smoothed fit to the data points.

We noted some small fluctuation in the concordance rates across multiple same-sample lane comparisons, particularly among lanes with a greater difference in total numbers of genotype calls (e.g. the occasional outlier in Figures 2A or B). To determine the extent to which the amount of data influences concordance rates of called genotypes, we performed two sets of comparisons between related and unrelated lanes by titrating sequenced reads. In the first set of comparisons, we kept the number of reads from one lane constant (at 140M reads) and varied the number of reads in the other lane in 20M read increments (Figure 3A). We also performed a series of comparisons whereby the number of reads was the same in both lanes, but varied between 40M and 140M reads, also in 20M read increments (Figure 3B). For the same-sample comparisons, the number of reads had a minor effect on the resulting concordance rate. However, the change in concordance rate was larger for the different-sample comparisons. As more reads were used, we were able to better distinguish the different-sample concordance rate from the same-sample concordance rate. However, even with 40M reads in at least one sample, the concordance rates are sufficiently different between same-sample vs. different-sample comparisons to accurately determine sample identity. We note that using more reads is preferable because it allows for increased resolution between the same-sample and different-sample comparisons.

## Discussion

Here we show that it is possible to determine if two lanes of data generated on the HiSeq 2000 are from the same individual. In addition to providing a straightforward gender prediction method, we also leverage the natural variation in the human population to uniquely “fingerprint” a sample from a subset of genotypes that can be accurately called. Using this detected variation, we can accurately distinguish samples based on concordance rates between lanes from the same sample versus lanes from different samples. This analysis is robust even with different read counts in the lanes being compared, and it can distinguish samples where partial consanguinity exists. Our approach is not limited to HiSeq 2000 data and can be applied to data generated on any high throughput platform. We have found this relatively straightforward method extremely useful in validating that lanes that are combined come from the same individual. It is potentially a particularly useful approach for genomes where polymorphic loci are not known. It might also be useful to identify lanes of data with differing/lower qualities, which would have a negative impact on downstream analyses. As high throughput sequencing becomes commoditized, such an approach will be increasingly important for validating large sequencing datasets.

## Methods

### Data generation

All data were generated on an Illumina HiSeq 2000 using standard library preparation and sequencing protocols provided by the manufacturer. These genomes have been sequenced for other on-going research projects, and the raw sequence data will be published in its entirety elsewhere for those purposes. Sample A is an unrelated male of Caucasian descent. Samples B and C are Caucasian male and female respectively with known partial consanguinity going back several generations (T. Markello, personal communication).

Processed data needed to reproduce our results here are provided below. 100 base reads that pass the Illumina chastity filter and contain at least 32 bases with Phred-scaled qualities Q20 or greater were aligned to the hg18 reference sequence using BWA [9] with default parameters. To make genotype calls, we only consider reads with mapping qualities of Q30 or greater and positions within the read with base qualities of Q20 or greater. See Table 1 for a summary of the data used in these analyses.

### Genotype calling

Genotypes on the autosomes were called with a Bayesian algorithm called MPG (for the Most Probable Genotype) [10] and only compared at positions where both lanes had an MPG score greater than or equal to 10 and had a confidence threshold, defined as the ratio of MPG to Q20-coverage, greater than or equal to 0.5. This latter filter scales the confidence measure of the genotype caller such that we require higher confidence at higher depths of coverage. We have found this eliminates a large fraction of erroneous genotype calls [11]. Concordance rates for each pair of lanes are provided as supplementary material at ftp://ftp.nhgri.nih.gov/pub/outgoing/elliott/plos_one/. The concordance rate between the lanes was calculated as the number of concordant positions that met the thresholds in both lanes, divided by the number of total positions that met the thresholds in both lanes. Genotype calls that did not meet the above-described thresholds were not considered in concordance calculations.

### Gender prediction

In order to easily determine if samples are male or female, we examined a number of genomes and identified representative regions on the X and Y chromosomes where coverage generally reflects the presence or absence of these chromosomes. Our methods for determining coverage, as well as the identity of these regions are provided in a shell script at ftp://ftp.nhgri.nih.gov/pub/outgoing/elliott/plos_one/.
